# Evaluating Closed Incision Negative Pressure Therapy Use Following High-Risk Caesarean Section in a Middle East Population

**DOI:** 10.7759/cureus.105500

**Published:** 2026-03-19

**Authors:** Haidar S Aal Mussa, Hatem Abdelhamid Abdelrahman, Hadeel Al-Khannaq, Lobna ElSayed Hamouda, Aishah Abdullah, Minimole Omana Amma, Alexa M Russell, Siobhan Lookess

**Affiliations:** 1 General Surgery, Dibba Hospital, Dibba Al-Fujairah, ARE; 2 Obstetrics and Gynaecology, Dibba Hospital, Dibba Al-Fujairah, ARE; 3 Global Health Economics and Outcomes Research, Solventum, Maplewood, USA

**Keywords:** caesarean section, negative pressure therapy, obesity, postoperative care, surgical wound infection, united arab emirates

## Abstract

Surgical site complications (SSCs) following caesarean section impact maternal morbidity and mortality. To mitigate SSC development, incision management strategies such as closed incision negative pressure therapy (ciNPT) can be utilised. The effect of ciNPT on the management of closed incisions following caesarean section was examined in the present study. Patients underwent caesarean section between 2022 and 2024 at a single acute care hospital in the United Arab Emirates (UAE). All 82 patients underwent vaginal cleansing and received antibiotics prior to surgery. A Pfannenstiel incision was used, followed by closure with subcuticular suturing. Patients received either traditional silver dressings (Mepilex Border Ag Dressing; Mölnlycke Health Care, Peachtree Corners, USA) (standard of care (SOC) group, n=28) or ciNPT (3M Prevena Incision Management System; Solventum, Maplewood, USA) (ciNPT group, n=54) for postoperative care. Patient and incision outcomes were assessed at each dressing change. Welch two-sample t-tests compared continuous variables, and Fisher's exact tests compared categorical variables. There were no significant differences between the ciNPT and SOC groups for baseline demographics; however, the ciNPT group had a higher proportion of severely obese patients (body mass index (BMI) >40 kg/m^2^) and patients who had two or more previous caesarean deliveries. The ciNPT group had a lower SSC rate compared to the SOC group (0/54 (0%) vs. 6/28 (21%), respectively; p=0.0011), as well as a lower number of surgical site infections (SSIs) (0/54 (0%) vs. 3/28 (11%); p=0.037) and deep infections (0/54 (0%) vs. 3/28 (11%); p=0.037). In this patient population, the use of ciNPT was well tolerated and may help improve clinical outcomes in high-risk patients undergoing caesarean delivery.

## Introduction

Rates of caesarean section have been increasing globally over the past decade. Data from 154 countries indicate that 21.1% of pregnant women undergo caesarean section, with rates projected to increase to over 50% by 2030 [1]. In 2018, data specific to western Asia (which includes the United Arab Emirates (UAE)) reported a caesarean section delivery rate of 31.7% [1]. This is similar to the 33% of caesarean section rate observed in tertiary care hospitals in Dubai in 2016 [2].

Caesarean section is an invasive procedure that carries a risk for surgical site complications (SSCs), including haemorrhage, haematoma, seroma, dehiscence, and surgical site infection (SSI). Patient and surgical factors can further increase the risk of SSC development. Increased maternal age, preeclampsia, premature rupture of membranes, multiple previous caesarean sections, obesity, diabetes, anaemia, immunosuppression, and hypertension have been reported to increase the risk of SSC following caesarean section [3-8]. Additionally, surgical procedure factors, such as the length of surgery, emergency procedure, and incision type, can also increase the risk of SSC development [7,9,10]. Rates of SSCs after caesarean section range between 0.08% and 30.4%, depending on the complication type, geographical region, and institution [11-15]. For SSI, recent estimates of the overall global incidence range from 5.0% to 8.0%, with a rate of 1.4% observed at a single institution in the UAE between 2016 and 2017 [6,9].

As SSCs can impact maternal outcomes and healthcare costs, postoperative incision management strategies have been used to mitigate post-surgical complications. Current postoperative standard of care (SOC) includes the use of post-surgical dressings, such as hydrocolloids, alginates, gauze, antibacterial dressings, silicone dressings, silver-containing dressings, and hydrogel dressings, depending on the needs of the individual patient and incision [7]. More recently, advanced wound care methods such as closed incision negative pressure therapy (ciNPT) have been utilised to help hold the incision edges together, act as a barrier against external contamination, decrease lateral tension on sutured/stapled incisions, remove fluids and infectious materials, and reduce oedema. However, limited evidence exists on the use of ciNPT over caesarean section incisions in the UAE. The effect of ciNPT in the management of closed incisions following caesarean section at a single acute care hospital in the UAE was examined in this study.

## Materials and methods

Patients underwent caesarean section between 2022 and 2024 at Dibba Hospital, Dibba Al-Fujairah, UAE. Retrospective data were obtained from a de-identified medical record review. At the time of the procedure, patients provided written consent for the use of de-identified data. This study was reviewed and approved by the Ministry of Health and Prevention Research Ethics Committee (MOHAP/DXB-REC/J.S.O/No.143/2025).

Inclusion criteria

All patients deemed high risk for postoperative complications were included in the analysis. High risk was defined as the presence of type 2 diabetes, a body mass index (BMI) >30 kg/m^2^ or <18 kg/m^2^, previous caesarean section, a history of wound infection, or prolonged or emergency caesarean section.

Exclusion criteria

Patients without pre-existing diabetes, a BMI between 18 kg/m^2^ and 30 kg/m^2^, no history of previous wound infection, and non-emergency caesarean section were excluded from the analysis.

Caesarean section

All 82 patients underwent vaginal cleansing and received intravenous antibiotics (cefazoline) prior to surgery. A single surgeon performed all caesarean section procedures. For each surgery, a Pfannenstiel incision was utilised, and incisions were closed using subcuticular suturing. Patients received either traditional postoperative dressings with silver (Mepilex Border Ag Dressing; Mölnlycke Health Care, Peachtree Corners, USA) or ciNPT (3M Prevena Incision Management System; Solventum, Maplewood, USA) for postoperative care. Patients were discharged from the hospital two to 10 days after surgery.

For patients who received traditional silver dressings (SOC group, n=28), the dressings remained in place for 3-7 days with dressing changes performed by post-acute care nurses. For patients who received ciNPT (ciNPT group, n=54), a 20-cm dressing (3M Prevena Peel and Place Dressing; Solventum, Maplewood, USA) was placed over the incision by a physician in the operating theatre following incision closure. Continuous negative pressure was initiated at -125 mmHg. Dressings were changed every 5-7 days and performed by a trained physician or wound care professional during a physician or wound clinic visit. 

All patient and incision outcomes were assessed at each dressing change. Additionally, data were collected on adverse events and complications related to the ciNPT device usage. Primary outcomes included the SSC assessment. SSC was defined as the presence of SSI (superficial, deep, or organ space), dehiscence, seroma, haematoma, cellulitis, or incisional serous discharge. Secondary outcomes assessed included length of stay (LOS) and number of emergency department visits for wound care.

Statistical analysis

Descriptive statistics were generated for all variables. Means and standard deviations (SDs) were calculated for continuous variables, while counts and frequencies were used for categorical variables. Welch two-sample t-tests were used to compare continuous variables. Fisher's exact tests were used to compare categorical variables. RStudio (R version 4.5.2, RStudio version 2026.01.0+392; Posit, Boston, USA) was used for all statistical analysis; a p-value <0.05 was considered statistically significant.

## Results

Patient demographics

There were no significant differences between the ciNPT and SOC groups in age, BMI, or previous caesarean delivery (Table 1). However, the ciNPT group had a higher proportion of severely obese patients (BMI >40 kg/m^2^) and patients who had two or more previous caesarean deliveries. The ciNPT group also had increased rates of pregestational hypertension (p=0.0245) and elective caesarean sections (p=0.0332) compared to the SOC group (Tables 1-2).

**Table 1:** Patient demographics BMI: body mass index; ciNPT: closed incision negative pressure therapy; N/A: not applicable; SD: standard deviation; SOC: standard of care The p-values in bold are statistically significant (<0.05).

Parameter	ciNPT (N=54)	SOC (N=28)	Statistical Test	Test Statistic	Degrees of Freedom	p-value
Age (years), mean ± SD	34.5 ± 4.5	32.5 ± 6.2	Welch two-sample t-test	1.49	41.9	0.1425
BMI (kg/m^2^), n (%)	-	-	Fisher's exact test	N/A	N/A	0.1523
Overweight (25-29.9)	3 (6%)	2 (7%)	-	-	-	-
Obese (30-39.9)	30 (56%)	21 (75%)	-	-	-	-
Severely obese (>40)	21 (39%)	5 (18%)	-	-	-	-
Previous caesarean delivery, n (%)	41 (76%)	21 (75%)	-	-	-	0.9999
Number of previous caesarean deliveries, n (%)	-	-	Fisher's exact test	N/A	N/A	0.2691
0	13 (24%)	7 (25%)	-	-	-	-
1	12 (22%)	10 (36%)	-	-	-	-
2	15 (28%)	3 (11%)	-	-	-	-
3	8 (15%)	6 (21%)	-	-	-	-
4	5 (9%)	1 (4%)	-	-	-	-
5	0 (0%)	1 (4%)	-	-	-	-
6	1 (2%)	0 (0 %)	-	-	-	-
Patient comorbidities, n (%)	-	-	-	-	-	-
Pregestational obesity	51 (94%)	24 (86%)	Fisher's exact test	N/A	N/A	0.2228
Pregestational diabetes	10 (19%)	4 (14%)	Fisher's exact test	N/A	N/A	0.7623
Pregestational pannus	3 (6%)	0 (0%)	Fisher's exact test	N/A	N/A	0.5476
Pregestational hypertension	9 (17%)	0 (0%)	Fisher's exact test	N/A	N/A	0.0245

**Table 2:** Obstetric comorbidities ciNPT: closed incision negative pressure therapy; SOC: standard of care Fisher's exact test was used for all categorical data. The p-values in bold are statistically significant (<0.05).

Parameter	ciNPT (N=54)	SOC (N=28)	p-value
Elective caesarean section, n (%)	37 (69%)	12 (43%)	0.0332
Gestational diabetes, n (%)	19 (35%)	15 (54%)	0.1614
Preeclampsia/eclampsia, n (%)	1 (2%)	0 (0%)	0.9999
Gestational hypertension, n (%)	1 (2%)	2 (7%)	0.2791
Failure to progress, n (%)	8 (15%)	5 (18%)	0.7559
Non-reassuring foetal heart tones, n (%)	2 (4%)	2 (7%)	0.6029
Breach/malpresentation, n (%)	8 (15%)	3 (11%)	0.741
Genital herpes simplex, n (%)	1 (2%)	0 (0%)	0.9999
Multiple gestation, n (%)	0 (0%)	1 (4%)	0.3415
Placenta previa/accreta, n (%)	4 (7%)	1 (4%)	0.6564
Failed forceps, n (%)	0 (0%)	1 (4%)	0.3415
Prior uterine surgery, n (%)	1 (2%)	0 (0%)	0.9999

Primary outcomes

**Figure 1:**
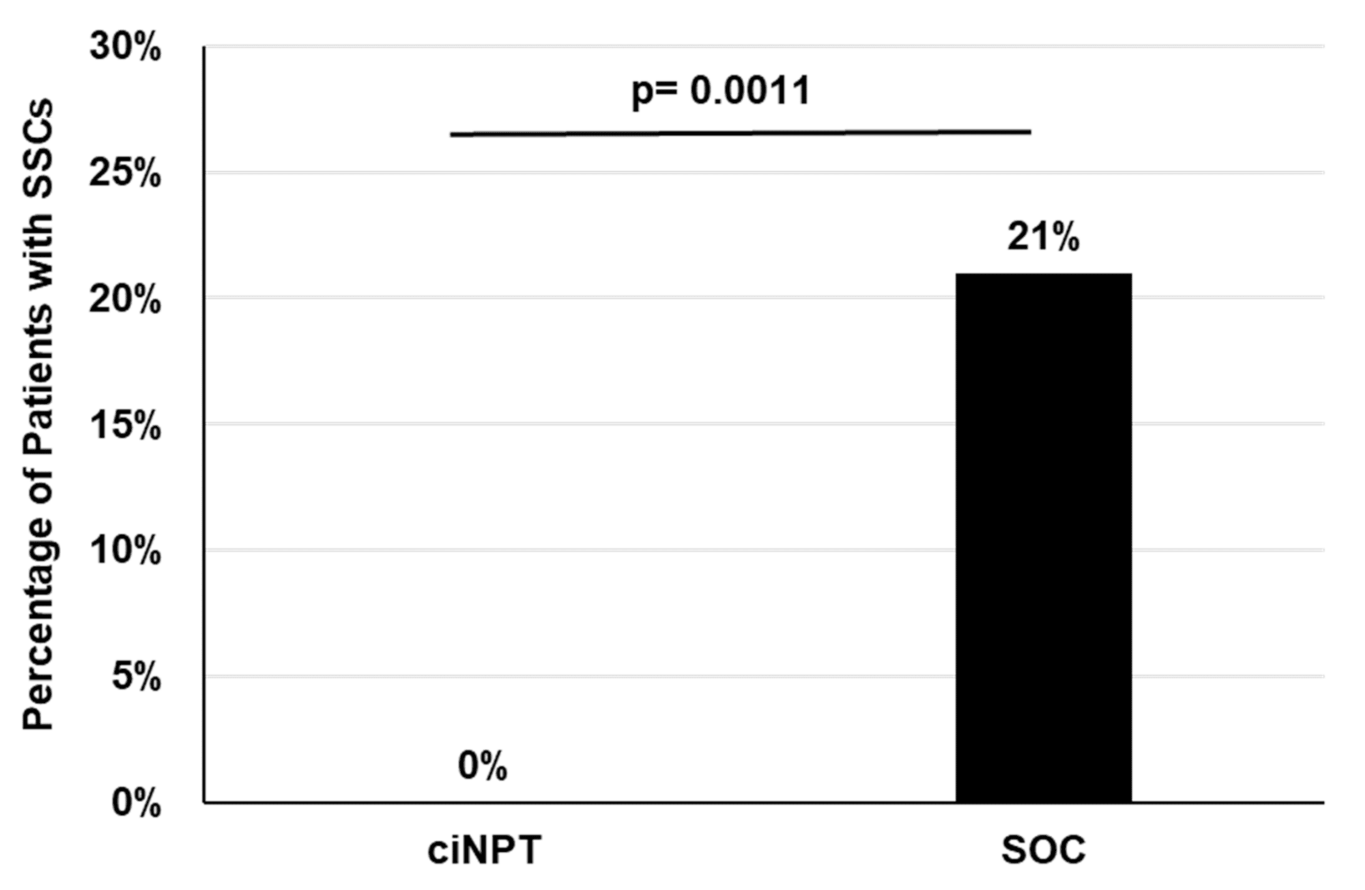
Percentage of patients with SSCs ciNPT: closed incision negative pressure therapy; SOC: standard of care; SSCs: surgical site complications

The ciNPT group had a significantly lower rate of SSC compared to the SOC group (p=0.0011) (Table 3, Figure 1). The ciNPT group also had a significantly lower rate of SSI compared to the SOC group (p=0.037) (Table 3). Additionally, all patients with SSI experienced a deep SSI. A clinical reduction in dehiscence rate was noted with ciNPT use, though this was not statistically significant (p=0.1138) (Table 3). None of the patients developed skin complications or adverse events related to negative pressure device use.

**Table 3:** Patient outcomes ciNPT: closed incision negative pressure therapy; N/A: not applicable; SD: standard deviation; SOC: standard of care The p-values in bold are statistically significant (<0.05).

Parameter	ciNPT (N=54)	SOC (N=28)	Statistical Test	Test Statistics	Degrees of Freedom	p-value
Surgical site complication, n (%)	0 (0%)	6 (21%)	Fisher's exact test	N/A	N/A	0.0011
Surgical site infection, n (%)	0 (0%)	3 (11%)	Fisher's exact test	N/A	N/A	0.037
Deep infection	0 (0%)	3 (11%)	Fisher's exact test	N/A	N/A	0.037
Dehiscence, n (%)	0 (0%)	2 (7%)	Fisher's exact test	N/A	N/A	0.1138
Serous discharge	0 (0%)	2 (7%)	Fisher's exact test	N/A	N/A	0.1138
Other	0 (0%)	1 (4%)	Fisher's exact test	N/A	N/A	0.3415
Time to discharge (days), mean ± SD	4.9 ± 1.4	4.8 ± 1.8	Welch two-sample t-test	0.26	43.8	0.7985
Emergency department visit for wound care, n (%)	0 (0%)	4 (14%)	Fisher's exact test	N/A	N/A	0.0117

Secondary outcomes

On average, both groups spent about five days in the hospital (Table 3). As these patients were at a high risk of developing SSC, they remained in the acute care setting for assessment and evaluation following caesarean delivery. However, there was a significant reduction in the number of emergency department visits for patients in the ciNPT group (p=0.0117) (Table 3).

## Discussion

A retrospective comparative study was performed using de-identified medical records from patients undergoing caesarean section, followed by the application of either ciNPT or postoperative dressing with silver for incision management. ciNPT use in these patients was associated with significantly reduced rates of SSCs and SSIs compared to post-surgical dressings with silver. The patient population included in this study was at high risk of developing SSCs due to increased BMI, multiple previous caesarean deliveries, diabetes, and hypertension. As such, the study authors selected ciNPT to help manage the incision and potentially mitigate SSC development.

ciNPT manages the incision by helping hold the incision edges together, acting as a barrier against external contamination, decreasing lateral tension on the incision, removing fluids and infectious materials, and reducing oedema [16,17]. These mechanisms of action have resulted in international guidelines recommending ciNPT use in patient populations at high risk of SSC development [18,19]. In these guidelines, ciNPT use is recommended for repeat incisions and individuals with two or more risk factors (such as BMI ≥30 kg/m^2^, diabetes, emergency procedures, and prolonged operation times) [18,19]. ciNPT use has been associated with reduced rates of SSCs and SSIs and shortened lengths of hospital stay across various surgical specialities [20]. However, the use of ciNPT following obstetric and gynaecological surgery has been associated with mixed results. 

Meta-analyses examining ciNPT use after caesarean section have reported similar SSC frequencies between ciNPT and control groups when examining overall SSC, which included dehiscence, seroma, and hospital readmissions [21-23]. For these meta-analyses, the patient population included a mix of normal-risk and high-risk patients, along with a mix of commercially available incisional negative pressure therapy devices. This could have impacted the overall clinical outcomes, as SSC rates are often low in normal-risk patients, and each negative pressure therapy device uses different therapy settings and dressing types. Three randomised controlled trials (RCTs) have also reported no differences in SSI, dehiscence, seroma, haematoma, LOS, readmission, or reoperation between ciNPT and control groups of patients at high risk of SSC development [24-26]. The Gunatilake et al. RCT examined the use of ciNPT following caesarean section in patients with high BMI and reported reduced rates of SSC, though these were not statistically significant [27]. Similarly, a retrospective cohort study noted similar rates of wound complications between ciNPT and traditional postoperative dressings after controlling for BMI and pregestational diabetes [28].

Conversely, there is a growing body of published clinical evidence that reports reduced rates of SSC associated with ciNPT use. Meta-analyses examining ciNPT use have reported reduced rates of SSI in the ciNPT groups compared to standard post-surgical dressings following caesarean section in both high-risk and normal-risk patient populations [29,30]. When examining overall SSCs, which included dehiscence, seroma, and hospital readmissions, Yu et al. reported reduced combined rates in the ciNPT group [29]. Arian and Kamali noted reduced rates of SSI in obese women with ciNPT use following caesarean section [30]. The Zhu et al. meta-analysis also reported reduced rates of SSI associated with ciNPT use following caesarean section in obese patients [31]. Importantly, these meta-analyses included a mix of negative pressure therapy devices rather than assessing the effect of one single ciNPT device. Previously published case/control studies using historical controls or retrospective data also noted reduced rates of wound complications and SSIs with ciNPT use in patients at high risk of post-surgical complications following caesarean deliveries [32,33]. These results mirror those reported in this study, where ciNPT use following caesarean section in patients with BMI >30 kg/m^2^ resulted in reduced rates of SSC and SSI when compared to postoperative dressings with silver. The reduction of SSC and SSI rates in this patient population may also improve the cost of patient care.

To assess the potential cost-saving effect of ciNPT use for this patient population, a hypothetical health economic model was developed to assess the direct costs related to surgical wound management (Table 4). This model examined the potential per-patient cost savings with ciNPT use for deep SSIs. As limited cost data for SSIs are available for the UAE, cost data in United States dollars (USD) were utilised. The potential cost of deep SSI was obtained from Hou et al., who examined the incidence of all SSI variations, including deep SSI, for open surgical procedures and the impact on LOS and costs attributable to SSIs for the index admission, readmissions, and outpatient visits [34]. The total patient cohort and the number of incidences of deep SSI for the model were obtained from the patient population in this study. For deep SSI, a potential cost of $20,522 per patient correlates to the mean total LOS and added costs of a deep SSI, illustrated in the Medicare cohort data from Hou et al., linked directly to the management of deep SSI in obstetrics and gynaecology patients in the United States (US) [34]. The total per-patient cost of therapy was included in the hypothetical cost model, with the SOC postoperative dressing monetary value being negligible due to its low unit cost. The model reported a total potential per-patient cost savings of $1,743 USD with the use of ciNPT compared to traditional postoperative dressings. This finding is similar to a study by Tuffaha et al., where a cost-utility analysis of ciNPT for high-risk caesarean section in Australia noted an incremental net monetary benefit of $70 (Australian dollars) compared to standard dressing use [35]. Similarly, a cost analysis using SSI rates following caesarean section from published literature reported that ciNPT use in patients at high risk of developing SSI could be cost-effective in groups with a potential SSI rate of >14%, such as patient populations with a BMI >45 kg/m^2^ [36].

**Table 4:** Hypothetical economic model ciNPT: closed incision negative pressure therapy; SSI: surgical site infection *Per patient therapy cost is estimated; individual prices may vary. This table is the authors' own creation.

Parameter	ciNPT	Silver Impregnated Dressing	Model Calculations
Number of patients	54	54	-
Number of deep SSI	0	3	-
Cost of deep SSI [34]	$20,522	$20,522	-
Per patient infection cost	$0	$2,199	(Number of deep SSI x cost per deep SSI) ÷ number of patients
Per patient therapy cost*	$495	$39	-
Total cost per patient	$495	$2,238	Cost per patient + per patient therapy cost
Potential per patient savings	$1,743	-	Total cost per patient ciNPT - total cost per patient silver impregnated dressing

Limitations

This study is limited by the retrospective design and the potential for selection bias. Only patients at high risk for postoperative complications were included in the study. The patients were then classified by postoperative care based on the de-identified medical record data. While no formal case-control matching occurred, baseline patient group demographics were similar, indicating that the analysis performed compared similar patient groups. The absence of SSC in the ciNPT group should be interpreted with caution due to the small sample size and the chance for effect overestimation, given the small number of events. While differences in clinical and/or operative factors could have contributed to these results, baseline characteristics between the groups were comparable. This indicates a potential effect on the reduction of SSC with ciNPT use that should be investigated more fully with larger patient populations. Additionally, the proposed hypothetical health economic model assessing the potential effect of ciNPT use on deep SSI was calculated using generic US health insurance data, USD, and complication rates from this study for a theoretical UAE population. Thus, the potential cost savings may be incorrectly estimated due to the lack of UAE-specific cost data.

To date, this study is the first to describe ciNPT use over caesarean section incisions in the UAE. ciNPT use over caesarean section incision data has been published for other global patient populations with mixed results, most likely due to patient population differences. This retrospective study was an initial look at this high-risk population. Future studies, including matched cohorts and RCTs, assessing ciNPT use within the UAE caesarean section population are needed to further evaluate the clinical and health economic outcomes in this population.

## Conclusions

High‑risk patients underwent caesarean delivery at a single acute care hospital in the UAE. In this cohort, ciNPT use was associated with reduced rates of SSCs, SSIs, and emergency department visits for wound care compared to standard postoperative dressings. These findings support the potential utility of ciNPT for incision management in patients at high risk for post-surgical complication development. However, the retrospective study design and the use of non‑UAE cost data for the hypothetical economic model limit these findings. Future prospective matched cohort studies and RCTs within the UAE are needed to fully evaluate both the clinical effectiveness and health economic impact of ciNPT in this patient population.
